# Longitudinal analysis of colon crypt stem cell dynamics in sulindac treated Familial Adenomatous Polyposis patients

**DOI:** 10.1038/s41598-017-11865-y

**Published:** 2017-09-20

**Authors:** Huiying Ma, Lodewijk A. A. Brosens, Sjoerd G. Elias, Folkert H. M. Morsink, Isaac J. Nijman, Linda M. Hylind, Elizabeth A. Montgomery, G. Johan A. Offerhaus, Francis M. Giardiello, Wendy W. J. de Leng

**Affiliations:** 1 Department of Pathology, University Medical Center Utrecht, Utrecht, The Netherlands; 2 Julius Center for Health Sciences and Primary Care, University Medical Center Utrecht, Utrecht, The Netherlands; 3 Department of Medical Genetics, University Medical Center Utrecht, Utrecht, The Netherlands; 4 Department of Medicine, The Johns Hopkins University School of Medicine, Baltimore, Maryland USA; 5 Department of Pathology, The Johns Hopkins University School of Medicine, Baltimore, Maryland USA

## Abstract

The non-steroidal anti-inflammatory drug sulindac decreases size and number of adenomas after 4-6 months of treatment for familial adenomatous polyposis (FAP) patients. However, the underlying mechanism remains unknown. As stem cells are thought to be the tumor precursor cells, visualizing their behavior is crucial for monitoring tumor progression. Increased tag diversity in inactive genes is indicative of a protracted clonal evolution and consequently, increased risk for tumor formation. Therefore, the effect of sulindac on stem cell dynamics was studied. Normal appearing single crypts were laser microdissected in placebo- and sulindac- treated FAP patient tissue after which the methylation patterns were visualized by Next Generation Sequencing. A significant difference in tag diversity over time was found in the sulindac group compared to the placebo group (*p = *0*.018), indicative of a shortened clonal evolution treated sulindac. The rate of change in tag diversity over time was correlated with polyp number change over time. No significant difference over time was observed in the percent methylation when comparing placebo vs sulindac. In conclusion, daily sulindac administration in FAP patients significantly altered colorectal stem cell dynamics, which might explain the chemopreventive action of this drug indicating that tag diversity may be used as a predictive biomarker.

## Introduction

In the intestine, differentiated cells are replaced every 5 days while stem cells can escape this fate^1^. For this reason, stem cells are believed to be the only cells that survive long enough to accumulate enough mutations to initiate tumorigenesis. Among stem cells, individual stem cell lineages compete for survival and replace other stem cell lineages, eventually resulting in a completely renewed stem cell pool. This continuous process in which a crypt is reconstituted from one single stem cell is called niche succession^2,3^. Since these stem cells are the tumor precursor cells, studying their behavior is important. However, no specific stem cell markers are available for routine diagnostics in human intestinal tissue and the tracing methods used in mouse models cannot be applied to humans. This led various scientists to investigate stem cell dynamics by measurement of the diversity of methylation patterns in the intestinal crypt^2,4,5^. Since methylation patterns are inherited through cell divisions during which methylation errors occur randomly, methylation patterns in inactive and therefore unregulated genes, can diverge over time. Using this method, Graham *et al*. found that less unique methylation patterns in a crypt indicated that this crypt had undergone a niche succession more recently compared to a crypt with a higher number of unique methylation patterns^6^. Baker *et al*. found a decreased methylation pattern diversity in adenomatous versus normal crypts in patients with familial adenomatous polyposis, indicating an increased stem cell loss/replacement rate in adenomas^5^. Pre-tumor progression is the phase during which there are no visible changes in the colorectal epithelium, but the cells nevertheless have already acquired early mutations for the initiation of tumor progression. The above methylation assay also allows for studying stem cell dynamics during pre-tumor progression^7^.

Familial adenomatous polyposis (FAP), a disorder characterized by the development of numerous colorectal adenomatous polyps between the age of 10 and 20 years^8^, has been used as an important model to study the transit from pre-tumor alterations to tumor progression^9^. FAP is an autosomal dominant hereditary disorder caused by a germline mutation in the Adenomatous Polyposis Coli (*APC*) gene. Additional mutations lead to a virtually 100% lifetime risk of colorectal carcinoma (CRC) development at a mean age of 39 years, only prevented by a prophylactic colectomy^10^. Other treatment strategies such as administrations of non-steroidal anti-inflammatory drugs (NSAIDs), namely sulindac and celecoxib, are effective in reducing both adenoma number and size^11,12^. In one study, sulindac intake of 300–400 mg per day resulted in a 71% reduction in adenoma formation compared to placebo after 4–6 months, a stronger treatment response than the average adenoma decrease of 23% in patients treated with 800 mg celecoxib a day for 6 months^12^. The number and size of adenomas increased immediately upon withdrawal of sulindac^13,14^. However, presently, the underlying mechanism of sulindac chemoprevention remains unclear.

A study on methylation pattern diversity revealed that stem cell niche succession occurs every 8.2 years in colorectal crypts of patients without FAP, whereas in FAP patients, stem cell lineage persists for an average of 32 years^15^. Our previous work on FAP patients also showed a significant increase in methylation pattern diversity in FAP patients compared to controls^16^. These results indicate that stem cells play a key role in the clinical course and complications of FAP. As sulindac treatment results in a decreased polyp size and number, we questioned whether sulindac treatment could influence stem cell dynamics, and, thereby, underlie the prevention or delay of polyp growth in FAP patients. Our previous study on stem cell dynamics used Sanger sequencing^16^ which is time consuming and therefore not suitable in a diagnostic is setting. Meanwhile, Next Generation Sequencing (NGS) has facilitated biomedical research and clinical diagnostics by allowing germline sequencing^17,18^, identification of novel genetic alterations^19^, identification of individual targeted therapy options^20,21^, methylation^22,23^ and stem cell dynamics^22–24^.

In the current study methylation patterns in FAP patients treated with sulindac were compared to placebo treated patients to study whether chemoprevention with sulindac is associated with changes in stem cell dynamics. Furthermore, methylation patterns were visualized with NGS and Sanger sequencing that was used as a technical control to validate the NGS workflow.

## Results

Diversity among stem cell lineages in the intestinal crypt can be investigated by analyzing changes in methylation patterns in the CpG island of the *CSX* gene. This gene is not expressed in the colorectum and changes in the methylation status are expected not to be regulated, i.e. they occur by chance. During cell division, CpG islands can randomly accumulate methylation errors^25^. Therefore, with ageing, methylation pattern diversity increases and drifts within and between individual crypts, which can be used to recapitulate crypt histories^4^.

To study stem cell dynamics, the diversity of unique methylation patterns per crypt and the percent methylation within a crypt were used^16^. The diversity of methylation patterns is defined as the total number of unique methylation patterns within a single crypt. The diversity reflects the genetic clonal diversity and time since all the stem cells went through a clonal homogenization event; i.e. duration of niche succession. Finding fewer patterns indicates a lower diversity; therefore the crypt is thought to have undergone a niche succession more recently. The percent methylation is defined as the number of methylated CpG sites compared to the total number of CpG sites. It reflects the mitotic age which is the total number of divisions since the zygote stage. Since methylation events accumulate randomly in unregulated genes during cell division, a higher percent methylation is indicative of a cell that has gone through more cell divisions.

Since the amount of methylation events increases with age, we carefully age matched four FAP patients treated with sulindac and four FAP patients treated with placebo (Table 1). All these patients were genotypically affected with familial adenomatous polyposis but phenotypically unaffected so they were quite young at the beginning of the study and no polyps were observed at the start of the study^14^. The polyp number increased with time in the placebo group and sulindac patients develop few polyps (Table 1, data from Giardiello *et al*.)^14^.

**Table 1:** Patient information.

Age (years)	Group	Gender	Race	Surgery	Polyp counts		
					0	4 months	2 years
8	sulindac	Male	White	None	0	0	0
9	sulindac	Male	White	None	0	0	0
9	sulindac	Female	White	None	0	1	0
14	sulindac	Female	White	None	0	0	5
8	placebo	Female	White	None	0	1	3
9	placebo	Male	White	None	0	1	10
14	placebo	Female	White	None	0	15	40
16	placebo	Female	White	None	0	4	7

**Figure 1:**
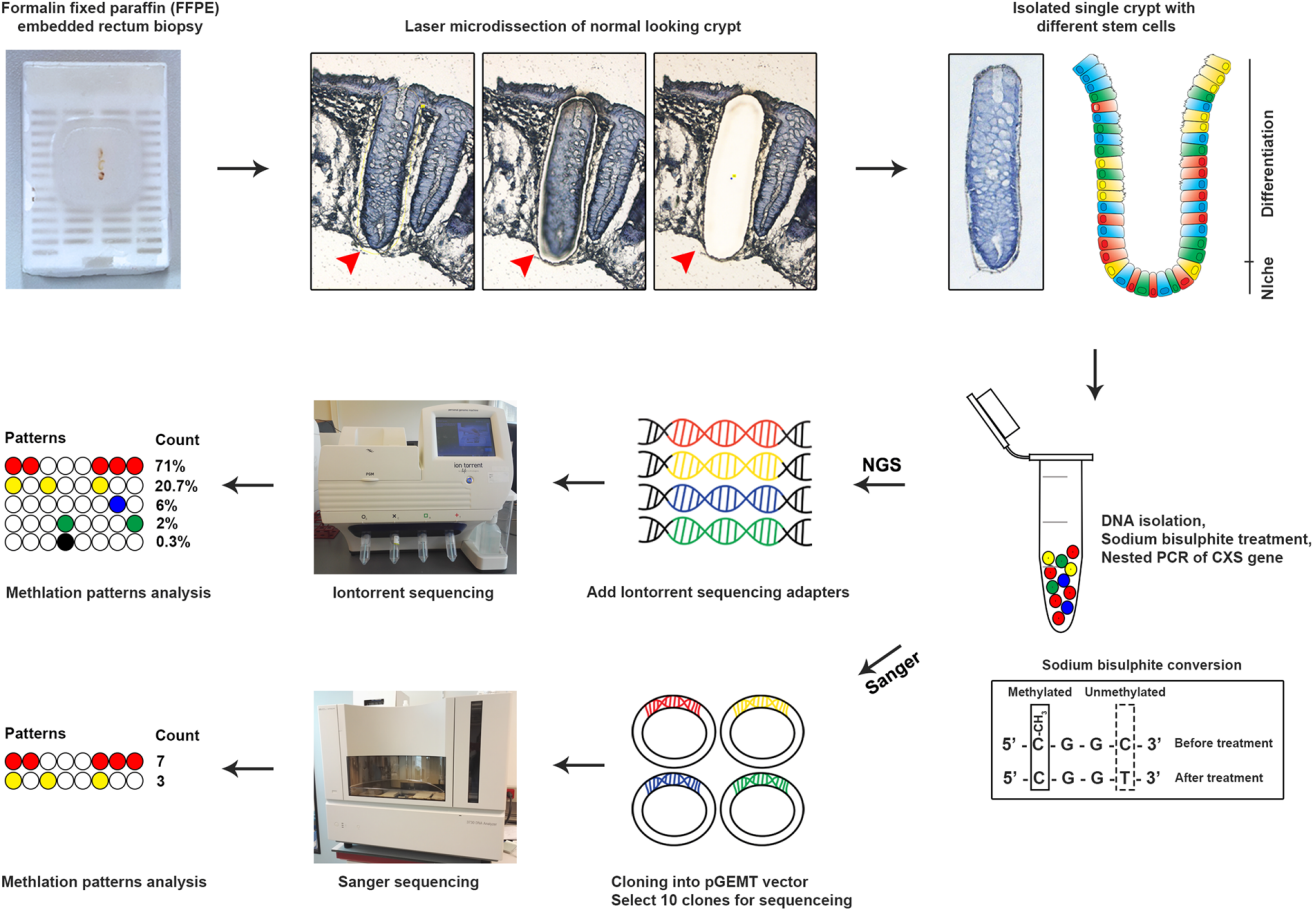
Workflow of stem cell dynamics assay. The assay is performed on archival paraffin embedded (FFPE) material from normal-looking tissues. Single crypts are laser microdissected, and DNA is isolated. Next, the extracted genomic DNA is treated with sodium bisulphite to convert unmethylated cytosines into uracil; methylated cytosines are protected from this turnover. A nested PCR is performed to target the *CSX* sequence. The PCR products are extended with Iontorrent PGM sequencing adapters and sequenced following the manufacturers protocol. The various methylation patterns per crypt can then be clustered by adapters. Different numbers of reads are found for each pattern including PCR errors which occurred below 1% count. Meanwhile, as a technical control, the same PCR products are cloned into bacterial vectors and enumerated after a sequence analysis of cloned inserts. 10 clones were selected for sequencing so for each crypt, the total number of counts are 10. CSX, cardiac-specific homeobox. Different colors represent different methylation patterns.

DNA from sulindac and placebo treated FAP patients at three different time points during treatment (t = 0, t = 4 m and t = 2 y) was laser microdissected from 10 normal appearing crypts for each patient. After sodium bisulfite treatment, PCR and sequencing, methylation patterns were determined. The workflow is shown in Fig. 1. The sequencing can be performed either by NGS or by Sanger sequencing, where with NGS more DNA molecules will be analyzed compared to Sanger sequencing. Furthermore, the Sanger approach contains an extra cloning step, which makes the NGS approach a higher throughput approach and therefore more convenient to study stem cell dynamics.

Using NGS, a great number of sequences ranging from 4 to 49360 reads (mean: 13656) were obtained per crypt (Supplementary Table S1). However, the vast majority of methylation patterns only appeared in a minority of the total number of sequencing reads. It was therefore assumed that these patterns were the result of PCR or sequencing errors, and, therefore, did not represent stem cell lineages within the crypts. A suitable threshold (cut-off point) was described for diagnostics for NGS data of FFPE tissue, where variants with an allele frequency lower than 1–5% were omitted from data analysis^26^. Therefore, we applied a 1% cut-off value for the final data analysis.

**Figure 2:**
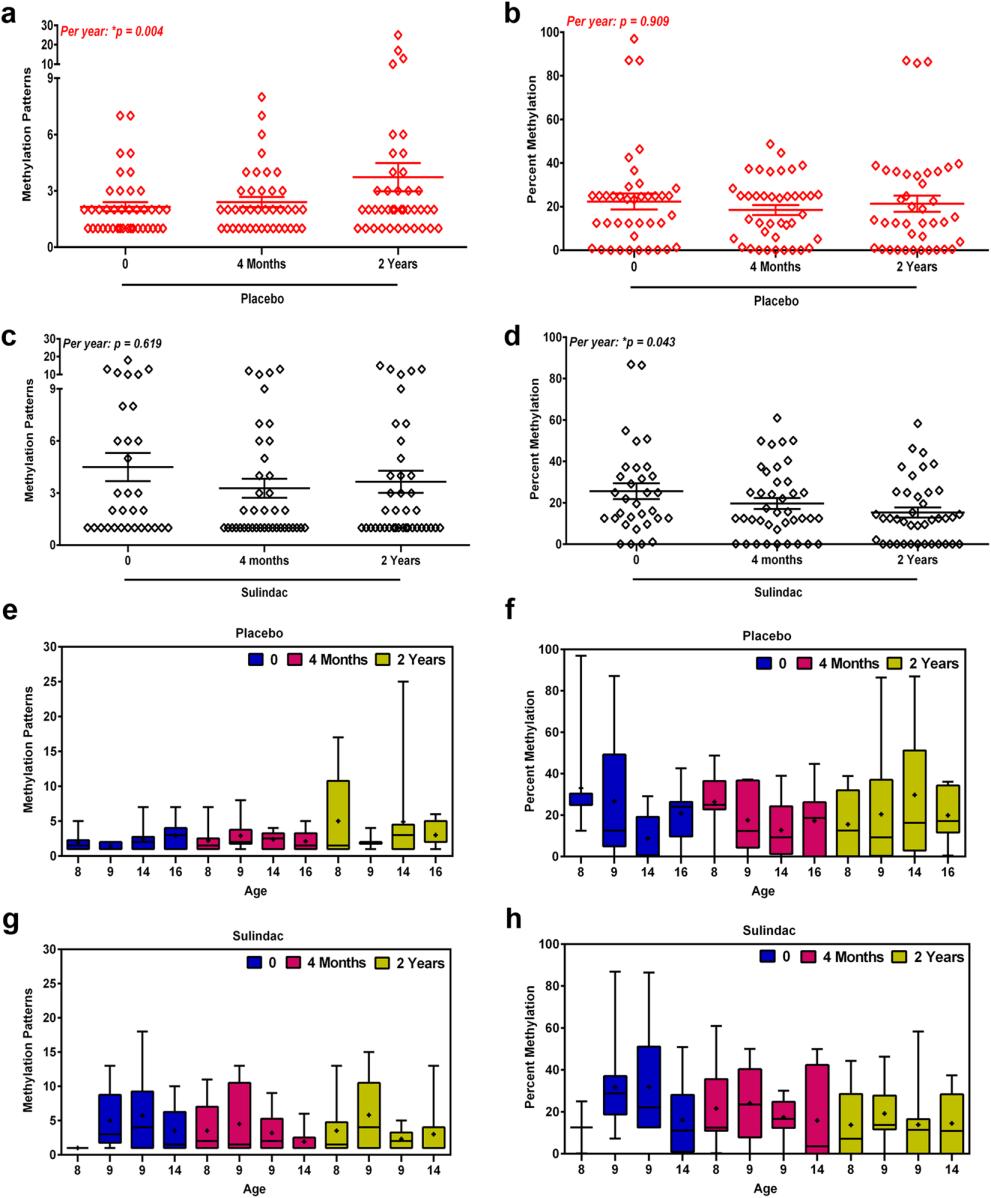
Stem cell dynamics analysis using NGS. (**a**,**b**) Methylation patterns and percent methylation of placebo group (red diamond) including time point 0, 4 months and 2 years; (**c**,**d**) Methylation patterns and percent methylation of sulindac group (black diamond) including time point 0, 4 months and 2 years; The trend in time per group (both placebo and sulindac) was indicated as p-values where other statistical values are mentioned in Table 2. (**e**,**f**) Methylation patterns and percent methylation for all four patients in placebo group; (**g**,**h**) Methylation patterns and percent methylation for all four patients in sulindac group. Each block represents one patient at indicated time point.

**Figure 3:**
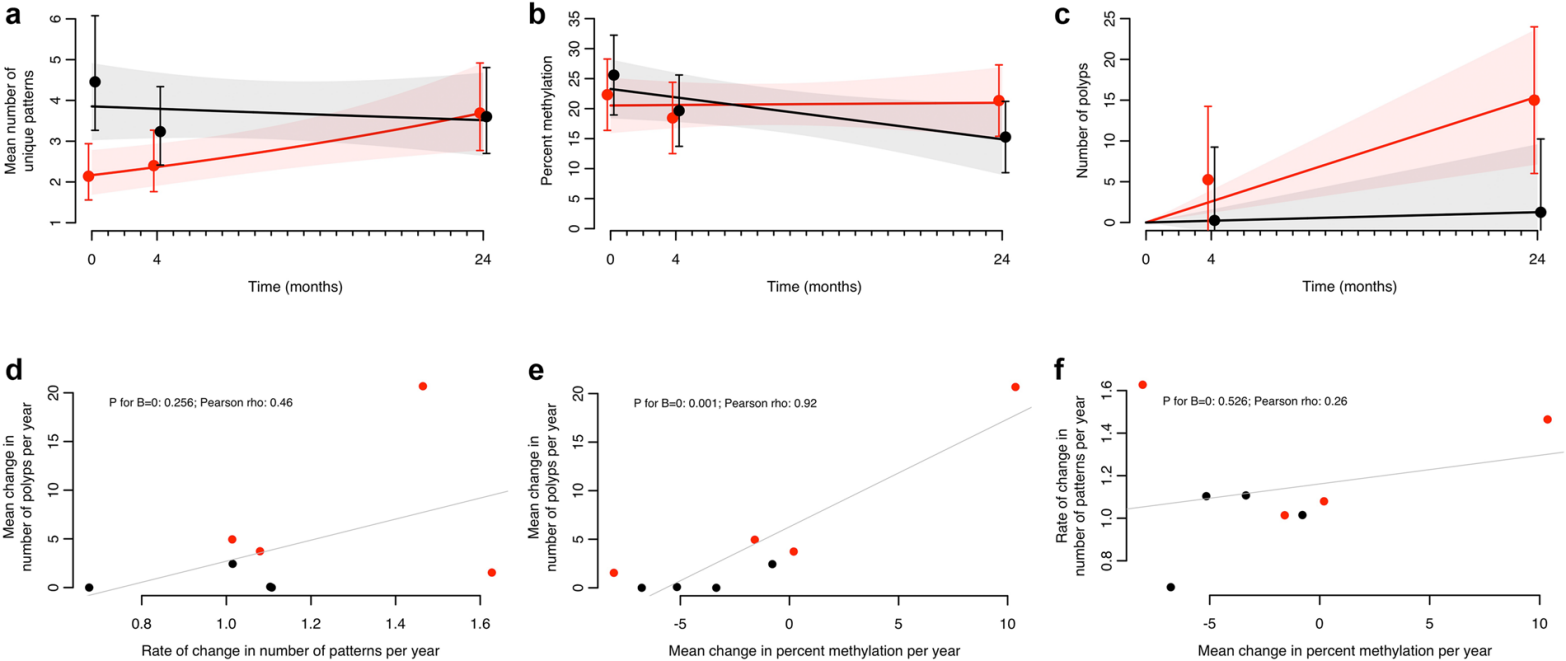
Multilevel mixed-effect regression analysis showing the interaction of treatment and time with methylation patterns and percent methylation together with polyp numbers. (**a**,**b** and **c**) Results are shown for methylation patterns (**a**), percent methylation (**b**) and number of polyps (**c**); the solid lines are the results from linear trend analyses; 95% confidence intervals are shown by error bars and the shadows surrounding the trend lines. (**d**–**f**) The relation between change per year in number of patterns (**d**) and percent methylation (**e**) with number of polyps, and each other (**f**) on an individual patient basis; lines show linear regression lines through data-points. Red: Placebo; black: Sulindac.

The effect of treatment on stem cell dynamics at the different time points was compared between the sulindac and the placebo group. Because the number of methylation patterns shows right skewness, we used mixed effect negative binomial regression that can handle count data and is robust for under- or overdispersion. In the placebo group a significant increase in the mean number of unique methylation patterns was observed over time (2.14 at t = 0, 2.40 at t = 4 m and 3.69 at t = 2 y; 4 m vs 0, *p* = *0*.*591*; 2 y vs 0, *p = *0*.*008*; 2 y vs 4 m, *p = *0*.*034*) resulting in an increased rate in the mean number of methylation patterns per year of 1.31 (95% CI 1.09–1.57; *p = *0*.*004*) when the trend with time was analyzed continuously. Contrarily, in the sulindac group no change was detected (4.46 at t = 0, 3.24 at t = 4 m and 3.60 at t = 2 y; 4 m vs 0, *p* = *0*.*118*; 2 y vs 0, *p* = *0*.*294*; 2 y vs 4 m, *p* = *0*.*583*) resulting in a rate in the mean number of methylation patterns per year of 0.96. (Table 2; Fig. 2a,c). A test for interaction showed that the trend with time in the number of methylation patterns significantly differed between the placebo and sulindac groups (*p = *0*.*018*; Table 2). The placebo group showed an increasing trend whereas the sulindac group remained constant with time, which is consistent with polyp development (Fig. 3a,c).

**Table 2:** Multilevel mixed-effect regression analysis of differences in the number of methylation patterns and percent methylation for sulindac and placebo treated FAP patients with time using NGS.

Group	Time point	Methylation Patterns			Percent Methylation		
Placebo		Mean	(95% CI)	p-value	Mean	(95% CI)	p-value
	0	2.14	(1.56;2.94)		22.3	(16.4;28.3)	
	4 months	2.40	(1.76;3.27)	0.591^$^	18.4	(12.5;24.4)	0.366^$^
	2 years	3.69	(2.77;4.92)	0.008^$^;0.034^&^	21.3	(15.4;27.3)	0.820^$^;0.500^&^
	Per year	1.31^#^	(1.09;1.57)	0.004	0.2	(−3.7;4.2)	0.909
Sulindac							
	0	4.46	(3.27;6.08)		25.6	(18.9;32.2)	
	4 months	3.24	(2.41;4.34)	0.118^$^	19.7	(13.7;25.6)	0.192^$^
	2 years	3.60	(2.70;4.81)	0.294^$^;0.583^&^	15.3	(9.3;21.2)	0.023^$^;0.306^&^
	Per year	0.96^#^	(0.80;1.14)	0.619	−4.2	(−8.2;−0.1)	0.043
Treatment*Time				0.018			0.125
Sulindac vs placebo							
	0			0.001			0.472
	4 months			0.170			0.778
	2 years			0.905			0.157

^$^Compared to time point 0; ^**&**^Compared to time point 4 months; ^**#**^Rate per year in the mean number of methylation patterns, this means that one has to multiply the mean number of patterns by the rate, taking the unit of time into account. For example: a rate of 0.96 (Table 2 sulindac patterns) means that if the mean number of patterns at T0 is 4.46, the mean number of patterns one year later will be 0.96*4.46 = 4.28, and again one year later it is 0.96*4.28 = 4.11.

Percent methylation was normally distributed and for that outcome we used mixed effect linear regression models to investigate the effect of treatment and time. The percent methylation in the placebo group remained constant over time (22.3% at t = 0, 18.4% at t = 4 m and 21.3% at t = 2 y; 4 m vs 0, *p* = *0*.*366*; 2 y vs 0, *p* = *0*.*820*; 2 y vs 4 m, *p* = *0*.*500*), with a mean change in percent methylation per year of 0.2% (95% CI −3.7 to 4.2%; *p* = *0*.*909*). For the sulindac group the percent methylation decreased from 25.6% at start of the study to 19.7% after 4 months and 15.3% after 2 years 4 m vs 0, *p* = *0*.*192*; 2 y vs 0, *p* = *0*.*023*; 2 y vs 4 m, *p* = *0*.*306*; Table 2; Fig. 2b,d) with a mean change per year of −4.2% (95% CI −2.8 to −0.1; *p = *0*.*043*). A test for interaction showed that the trend with time in percent methylation was not significantly different between both groups (*p* = *0*.*125*; Table 2). The placebo group remained constant whereas the sulindac group slightly decreased trend with time (Fig. 3b).

Although individual results per patient for the sulindac and placebo groups showed no age related differences for the individual patients (Fig. 2e–h), there is a slight baseline difference in age between the placebo and the sulindac group (11.5 vs 9.75 years) which cannot be explained by above mentioned results (Table 2 and Fig. 3a). Therefore, we analyzed the data for treatment and time in relation to methylation patterns and percent methylation which remained unaffected after adjustment for age in the mixed-effects models (Supplementary Table S2).

We also estimated per patient the rate of change in mean number of methylation patterns, the change in mean percent methylation and the change in number of polyps over time, and then related these individual estimates of stem cell dynamics with polyp development and with each other (Fig. 3d–f). These results show a positive correlation between the three phenomena, indicating that patients in whom the number of methylation patterns (Fig. 3d) or the percent methylation (Fig. 3e) increases more in time also develop more polyps, and that in patients in whom the number of methylation patterns increase more in time also show more increase in percent methylation in time (Fig. 3f), and *vice versa*.

As sequencing depth might influence the results, the data were re-analyzed using only samples with ≥1000x sequencing. This sensitivity analysis, where 194 of the 232 crypts remained for the analysis, also showed similar results (Supplementary Table S3). As a technical validation, the same DNA samples were also analyzed using sanger sequencing (where 10 clones were sequenced per crypt), resulting in a concordance rate of at least 96% (Supplementary information; Supplementary Table S4 and Supplementary Fig. S1).

## Discussion

Sulindac treatment effectively induced colorectal polyp regression in FAP patients after 3–6 months, although it did not prevent the development of new polyps in pre-symptomatic FAP patients^14^. This short-lived effect of sulindac on polyp regression might be due to increased prostaglandin levels^14^, retained nuclear accumulation of β-catenin and reduced epithelial COX-2 expression^27,28^ or the occurrence of mutations such as in *KRAS* ^12,29^. Here we studied whether the short beneficial effect of sulindac treatment is associated with changes in stem cell behavior. Stem cells can acquire somatic mutations, which are either silent or genetically advantageous. These mutations are passed on to daughter cells and may provide a selection force to a stem cell lineage, leading to a stronger dominance and a subsequently higher longevity of the stem cell lineage^30^. If a stem cell lineage persists longer, the time to visible tumor progression decreases, because neoplastic mutations can accumulate in the stem cells^2,31^. Also, in the case of FAP, a heterozygous mutation in *APC* may enhance the probability of a symmetrical stem cell division, leading to a larger stem cell pool^32^. As mentioned, niche succession in FAP takes considerably longer than in the normal colon and consequently the risk of an increased mutational load is higher. This has also been suggested by Kim *et al*., who found an increased stem cell survival in FAP patients compared to controls^15^. An increased number of stem cells in the colorectal crypt is associated with an expansion of the proliferative compartment towards the epithelial surface, which further enhances the risk of relevant carcinogenic alterations^33^.

The first study on the role of methylation patterns in SC dynamics used Sanger sequencing, the “gold standard” sequencing technique. Despite its accuracy, this method is labor intensive, relatively slow, and has a low throughput^34^. Furthermore, to identify methylation patterns for individual stem cells, a cloning approach is necessary, making the assay particularly laborious. Recently, NGS has emerged as a high throughput technique that provides reliable sequence data even from sub-optimal starting material like formalin fixed paraffin embedded (FFPE) tissues^35^. A clonal amplification of each sequencing read is performed as an intrinsic component of the NGS process such that additional cloning steps to identify individual stem cells are bypassed. We detected 96% similarity in methylation events comparing NGS to Sanger sequencing. As expected, due to the higher sensitivity of NGS, more sequences and thereby more methylation patterns were found using NGS analysis. Although errors occur in NGS, a methylation pattern frequency cutoff of 1% adjusted for this. Hence, NGS can not only substitute for Sanger sequencing in studying stem cell dynamics, but also provide additional data.

When comparing the unique number of methylation patterns in the placebo versus the sulindac-treated FAP patients a significant increase is visible after 2 years of treatment in the placebo group. In contrast, a slight decrease was found in the sulindac group, especially after 4 months of treatment. Four months coincides with the time when polyp number is still repressed and resistance only starts to develop after 6 months^13^. There is a difference between the sulindac and placebo group for the number of methylation patterns at baseline. This occurred by chance and is unfortunate, but the difference in trend between the two groups is striking and is consistent with diminished stem cell lineage longevity due to sulindac (Table 2). It decreases the chance of accumulated mutations and corresponds with the lower number of polyps in the treatment group^14,36,37^.

Also a significant difference was found in sulindac treated patients with regard to mitotic age, represented by the percent methylation^25^. A higher mitotic age corresponds to a higher number of cell divisions that a stem cell has undergone and is associated with an increased risk of accumulating mutations. We found a slight decrease in the percent methylation between the start of the study and after 4 months of sulindac treatment, and a significant decrease in percent methylation between the start of the study and 2 years of sulindac administration, whereas the placebo group remained at a similar level. The lower percent methylation after 4 months of sulindac treatment corresponds with the lower polyp count observed in previous studies^12–14^. The effect, however, seems temporary^14^, and apparently other pathways can be used to circumvent the growth inhibition^27,29,38^. In the sulindac group, a borderline decrease was observed per year. However, there was no significant difference after treatment over time which might be a reason why this effect is temporary.

In the current study archival FFPE tissue was used which results in crosslinking of the DNA and artefacts like deamination of cytosines^39^. Although the deamination might influence the methylation patterns described in this study, it will affect both study groups equally. It will result in changes from a cytosine to uracil, thereby underestimating the amount of methylation. When comparing our numbers to studies that used fresh frozen tissue this seems indeed the case^6,15,40^. The sulindac and placebo treated patients were age matched in our study and a standardized biopsy site was used, thereby minimizing any bias. This precluded incorporation of a control group of normal subjects in our study. Location in the colorectum and also age are determinants of methylation and therefore confounders in a study like this. We also realize that the numbers are small and that conclusions need to be drawn with caution. However, it is felt that our study suggests that stem cell behavior may indeed be influenced by sulindac.

In summary, we provide evidence that NGS can be applied accurately and efficiently in studying stem cell dynamics. Furthermore, daily sulindac administration significantly altered colorectal stem cell dynamics over time, consistent with decreased stem cell lineage longevity. This effect may contribute to the observed inhibition of polyp numbers in sulindac treated FAP patients.

## Material and Methods

### Patients

Biopsy materials from eight FAP patients, ranging from the age of 7 to 15 years (Table 1), were selected from the study population of Giardiello *et al*.^14^. All materials are archival tissues of anonymized patients from Johns Hopkins Polyposis Registry that were used. Written informed consent was obtained from all subjects or their parents, and consent was obtained from subjects under 18 years of age. The protocol was approved by the Johns Hopkins Joint Committee on Clinical Investigation (the institutional review board). All patients had no detectable polyps at the start of the study and received 75 mg (body weight between 20 and 44 kg) or 150 mg (body weight more than 44 kg) sulindac or placebo orally twice a day. Before sulindac or placebo administration, colorectal biopsies were taken from the normal appearing mucosa (t = 0), and also after 4 months (t = 4 m) and 2 years (t = 2 y) after the start of treatment. The biopsy sites were standardized and taken from the rectum-flat mucosa. Biopsies were formalin-fixed and embedded in paraffin. The research was carried out in accordance with the ethical guidelines of the research review committee of our institution.

### DNA isolation from single crypt

Paraffin embedded tissue was cut into 10 μm P.A.L.M. slides which were especially for laser micro-dissection microscopy. The slides were deparaffinized and counterstained with haematoxylin. 10 unique, normal appearing longitudinal crypts were isolated using a P.A.L.M. (Zeiss) system, and incubated with 20 μL PicoPure extraction solution (PicoPure DNA Extraction Kit, MDS Analytical Technologies) according to the manufacturer’s instructions.

### Sodium bisulphite conversion and nested PCR

DNA was treated with sodium bisulphite (EpiTect Bisulphite Kit, Qiagen) according to the manufacturer’s instructions. Sodium bisulphite converted DNA was used in a nested PCR to amplify a CpG island containing 8 CpG sites in the *CSX* gene. This gene is not expressed in the colon. The first PCR reaction mix had a total volume of 24 µL including 12 µL KAPA Hifi Uracil+ Ready mix (Kapa biosystems), 2 µL of each primer at 2 µM (For 5′-GGGGAGAAGGGGTTTTTAATAT-3′ and Rev 5′-AAAAACACTCCTAAAAAAACAACTAA-3′), and 8 µL of template DNA. PCR1 program consisted of an initial denaturation at 95 °C for 2 minutes, followed by 40 cycles of denaturation at 98 °C for 20 seconds, primer annealing at 61 °C for 15 seconds, extension at 72 °C for 30 seconds, and a final extension step at 72 °C for 1 minute. The second PCR reaction mix had a total volume of 20 µL comprising of 4 µL KapaHifi Buffer (5X), 0.4 µL MgCl2 (25 mM), 0.6 µL dNTPs (10 mM), 0.5 µL of each respective primer at 10 mM (For 5′-GTAAAACGACGGCCAGGGAGATTTAGGAATTTTTTTTGTTTT-3′ and Rev 5′-CAGGAAACAGCTATGACACACCAAACTACAAAATCACTCATTA-3′), 0.2 µL KAPA Hifi Hotstart (1 U/µL), and 1 µl of DNA template taken from PCR 1 (diluted 1:100). PCR2 program consisted of an initial denaturation at 98 °C for 45 seconds, followed by 34 cycles of denaturation at 98 °C for 20 seconds, primer annealing at 58 °C for 15 seconds, extension at 72 °C for 30 seconds, and a final extension step at 72 °C for 1 minute.

### Next Generation Sequencing – Ion torrent

The other half of the volume of the nested PCR products was used for NGS. To sequence these amplified segments of the *CSX* gene specialized sequencing adapters need to be attached to the amplicon, which was accomplished by a fusion PCR. Furthermore, barcodes were attached to each individual sample so that they can be identified after sequencing (see Supplementary Table S5 for primer sequences). The fusion PCR was carried out in a total volume of 20 µL comprising of 4 µL KAPA Hifi Buffer (5X), 0.4 µL MgCl2 (25 mM), 0.6 µL dNTPs (10 mM), 5 µL of each respective primer at 10 µM, 0.2 µL KAPA Hifi Hotstart (1 U/µL), and 1 µl of DNA template taken from PCR 2 (diluted 1:100). The fusion PCR reaction program: initial denaturation at 98 °C for 45 seconds, followed by 34 cycles of denaturation at 98 °C for 20 seconds, primer annealing at 58 °C for 15 seconds, extension at 72 °C for 30 seconds, and a final extension step at 72 °C for 1 minute. Next, the DNA was purified using the Agencourt® Ampure® XP kit according to the manufacturer’s instructions. All the samples were pooled at a final concentration of 0.8 pg/µL and sequenced on the Ion Torrent PGM (Thermo Fisher) using the Ion PGM™ 200 bp kit and the Ion 318™ Chip v2.

### Sanger sequencing

Half of the volume of the nested PCR products was purified with the Illustra GFX PCR DNA and Gel Band Purification Kit (GE Healthcare) according to the manufacturer’s instructions. PCR products were eluted in 30 μL Elution Buffer and incubated for 10 min at 72 °C with NEB DNA polymerase to create an A-overhang. This solution contained 0.2 mM dNTPs, 2.0 mM MgCl_2_, 0.75 U NEB DNA polymerase (New England Biolabs), with an end concentration of 1x NEB DNA polymerase PCR buffer (New England Biolabs). PCR products were then cloned using the pGEM-T Easy Vector System (Promega) into competent *E. coli* Top10 cells. Successfully cloned inserts were amplified in a colony PCR. PCR products were incubated with the ABI Big Dye Terminator Mix (Applied Biosystems) and run on an ABI 3730 genetic analyzer (Applied Biosystems) for sequencing. Sequences were analyzed with DNASTAR lasergene 12.2, and sequences with incomplete bisulphite conversions were excluded from analysis.

### Data analysis

For each patient, 10 normal appearing crypts were isolated and for each crypt, 10 sequences were used for Sanger analysis, each containing methylation information of 8 CpG sites. IonTorrent reads were assigned to samples by custom scripts reading the internal barcoding tags. Since the reads were from a single amplicon, they were aligned using BWA^41^ using default parameters to an in-silico converted reference sequence resembling a fully methylated, bisulphite converted region. Only reads were kept if they fully covered the amplicon. CpG positions (2, 4, 6, 18, 22, 37, 40 and 51) and control positions (14, 20, 14) were genotyped within the context of a single read with a custom walker for GATK^42^. A custom per script was used to collect all reads with a perfect conversion at the control sites and to score all the various combinations and percent methylation at the CpG sites. Scripts are available upon request. The number of unique methylation patterns was determined for each crypt, and the percent methylation was determined by comparing the number of methylated CpG sites to the total number of CpG sites per crypt^6,16^.

We used multilevel mixed-effects regression models to study the relation between time and treatment with stem cell dynamics, using the individual crypt derived data-points as the unit of analysis while taking within-patient clustering into account with random intercepts. For the number of methylation patterns – i.e. count data – we used mixed-effects negative binomial regression, with which we estimated the mean number of patterns per treatment group per time-point if time was analyzed categorically, and the rate of change in mean number of patterns per year for each treatment group if time was analyzed continuously (i.e. as a trend). For the percent methylation – i.e. a normally distributed continuous outcome – we used mixed-effects linear regression, with which we estimated the mean percent methylation per treatment group per time-point if time was analyzed categorically, and the mean change in percent methylation per year for each treatment group if time was analyzed continuously (i.e. as a trend). All these models included treatment and time as main effects as well as their interaction. Model assumptions were checked and were not violated. All estimates are reported with their corresponding 95% CIs. To assess the relation between changes in percent methylation, methylation patterns, and polyp development with time per patient, we estimated per patient the mean change in percent methylation by linear regression, the rate of change in methylation patterns by negative binomial regression, and the change in number of polyps by linear regression over time (all linearly), and then related these individual estimates of methylation and polyp development dynamics with each other by scatterplots, linear regression, and estimating Pearson correlation coefficients.

R version 3.2.1 was used for statistical analysis (especially using lme4) and figures were made in GraphPad Prism 6. Statistics method was indicated in the legends and applied for analysis and statistical significance was defined as *p < 0.05. The means ± SEM were displayed in the figures where applicable.

### Data Availability

The data concerning the number of polyps are derived from the previous paper by Giardiello, F. M. *et al*.^14^. The remaining data generated during this study are included in this article (and its supplementary information files). The original NGS data is included as Supplementary Table S6 and BAM files are available upon request.

## Electronic supplementary material

Supplementary information

Supplementary Table S6
