# Supplementary material for: Longitudinal analysis of colon crypt stem cell dynamics in sulindac treated Familial Adenomatous Polyposis patients
Source: Sci Rep. 2017 Sep 20;7:11972. doi: 10.1038/s41598-017-11865-y (PMC5607292; doi:10.1038/s41598-017-11865-y)
Supplement: Supplementary file 1 — Supplementary information [file 41598_2017_11865_MOESM1_ESM.pdf]

Longitudinal analysis of colon crypt stem cell dynamics in sulindac treated Familial Adenomatous Polyposis patients

Huiying Ma[1], Lodewijk A.A. Brosens[1], Sjoerd G. Elias[2], Folkert H.M. Morsink[1], Isaac J. Nijman[3], Linda M. Hylind[4], Elizabeth A. Montgomery[5], G. Johan A. Offerhaus[1], Francis M. Giardiello[4], Wendy W.J. de Leng[1*]

[1] Department of Pathology, University Medical Centre, Utrecht, the Netherlands

[2] Julius Center for Health Sciences and Primary Care, University Medical Center Utrecht

[3] Department of Medical Genetics, University Medical Centre, Utrecht, the Netherlands

[4] Department of Medicine, The Johns Hopkins University School of Medicine, Baltimore, Maryland, USA

[5] Department of Pathology, The Johns Hopkins University School of Medicine, Baltimore, Maryland, USA

[*] Corresponding author:

Wendy W.J. de Leng, Department of Pathology, University Medical Center Utrecht, 3584 CX Utrecht, The Netherlands; Tel: +31-887553610; E-mail: W.W.J.deLeng@umcutrecht.nl

Supplementary information

Supplementary results

SC dynamics analysis using Sanger sequencing results

Due to the limitation of Sanger sequencing, in total only 10 sequences could be obtained. For the placebo group, a slight increase was detected in the unique number of methylation patterns (1.38 at t=0, 1.40 at t=4m and 2.08 at t=2y), whereas the percent methylation remained unchanged in time (23% at t=0, 18% at t=4m and 22% at t=2y) (**Supplementary Fig. S1a and b**). Within the sulindac group, a slight decrease in the unique number of methylation patterns (2.09 at t=0, 1.75 at t=4m and 1.69 at t=2y) and the percent methylation (27% at t=0, 22% at t=4m and 20% at t=2y) was observed after 2 years of sulindac treatment, compared to the start of treatment. Due to limited tissue availability only 2 crypts could be analyzed for one patient (aged 8 years) (**Supplementary Fig. S1c and d**). When looking at the individual patients, three patients (9, 9, 14 years-old) in the sulindac group showed a decrease in the number of unique methylation patterns after 4 months, however, after 2 years, an increase was found (**Supplementary Fig. S1e, f, g and h**).

To determine whether NGS can replace Sanger sequencing to visualize stem cell dynamics, results for the number of unique methylation patterns and percent methylation of the two methods were compared (**Supplementary Table S4**). For the methylation pattern-based comparison, the total number of unique methylation patterns was determined for the Sanger and the NGS method, after which the similarity was calculated as the number of similar patterns divided by the total number of patterns identified using Sanger sequencing. For the placebo group this resulted in a similarity of 97% and for the sulindac group of 98%. For the crypt-based comparison, the total number of unique methylation patters per crypt was analyzed, where the results was considered similar between the two methods when all 10 patterns identified using Sanger sequencing were also identified using NGS.

Both for sulindac and placebo group 5 out of respectively 107 and 115 crypts were found having different sequences resulting in a similarity of 96% and 96%, respectively.

Supplementary figure legend

Supplementary Figure S1 Methylation patterns and percent methylation results of Sanger sequencing. a and b Methylation patterns and percent methylation of placebo group including time point 0, 4 months and 2 years; **c and d** Methylation patterns and percent methylation of sulindac group including time point 0, 4 months and 2 years; **e and f** Methylation patterns and percent methylation for all four patients in placebo group; **g and h** Methylation patterns and percent methylation for all four patients in sulindac group.

Supplementary Table S1. Sequencing depth for NGS

Group	Time point	Sequencing depth		
**Placebo**		**Mean**	**Median**	**Minimum;Maximum**
	0	13927	12737	104; 31038
	4 months	13626	13543	19; 49360
	2 years	9919	8268	55; 26769
**Sulindac**				
	0	17116	20053	35; 25900
	4 months	13736	15776	4; 27606
	2 years	14302	17091	6; 24722

Supplementary Table S2 Multilevel mixed-effect regression analysis of differences in the number of methylation patterns and percent methylation for sulindac and placebo treated FAP patients using NGS (Adjusted by age)

Group	Time point	Methylation Patterns			Percent Methylation		
**Placebo**		**Mean**	**(95%CI)**	**p-value**	**Mean**	**(95%CI)**	**p-value**
	0	2.17	(1.58;2.98)		23	(17.0;28.9)	
	4 months	2.42	(1.78;3.30)	0.606$	19.1	(13.2;25.0)	0.363$
	2 years	3.73	(2.80;4.97)	0.008$;0.033&	22	(16.1;27.9)	0.818$;0.496&
	Per year	1.31#	(1.09;1.57)	0.005	0.2	(-3.7;4.1)	0.909
**Sulindac**							
	0	4.43	(3.25;6.04)		25.3	(18.7;31.9)	
	4 months	3.19	(2.37;4.29)	0.109$	18.8	(12.8;24.8)	0.149$
	2 years	3.56	(2.66;4.76)	0.281$;0.576&	14.4	(8.4;20.4)	0.016$;0.302&
	Per year	0.95#	(0.80;1.14)	0.609	-4.4	(-8.4;-0.3)	0.034
**Treatment*Time**				0.017			0.109
**Sulindac vs placebo**							
	0			0.002			0.604
	4 months			0.211			0.944
	2 years			0.824			0.079

Rate per year in the mean number of methylation patterns; $ Compared to time point 0; & Compared to time point 4 months.

Supplementary Table S3 Multilevel mixed-effect regression analysis of differences in the number of methylation patterns and percent methylation for sulindac and placebo treated FAP patients using NGS (Coverage ≥ 1000x sequencing)

Group	Time point	Methylation Patterns			Percent Methylation		
**Placebo**		**Mean**	**(95%CI)**	**p-value**	**Mean**	**(95%CI)**	**p-value**
	0	2.02	(1.42;2.88)		21.7	(15.5;27.9)	
	4 months	2.17	(1.51;3.10)	0.771[$]	16.6	(10.2;22.9)	0.258[$]
	2 years	3.79	(2.71;5.29)	0.007[$];0.017[&]	22.1	(15.6;28.7)	0.924[$];0.233[&]
	Per year	1.38[#]	(1.12;1.71)	0.003	1.2	(-3.1;5.4)	0.596
**Sulindac**							
	0	4.22	(2.99;5.98)		24.1	(17.3;31.0)	
	4 months	3.09	(2.18;4.39)	0.185[$]	15.3	(8.6;21.9)	0.069[$]
	2 years	3.17	(2.24;4.47)	0.214[$];0.922[&]	11.2	(4.6;17.7)	0.007[$];0.391[&]
	Per year	0.91[#]	(0.74;1.12)	0.378	-5.1	(-9.5;-0.7)	0.024
**Treatment*Time**				0.006			0.047
**Sulindac vs placebo**							
	0			0.003			0.604
	4 months			0.162			0.779
	2 years			0.462			0.02

[#] Rate per year in the mean number of methylation patterns; [$] Compared to time point 0; [&] Compared to time point 4 months.

Supplementary Table S4. Comparison between Sanger sequencing and NGS

Method	Group	Sequence method	Total pattern number	Same pattern number	Similarity[#]
**pattern-based**	sulindac	Sanger	204	199	97.55%
		NGS	2227		
	placebo	Sanger	194	188	96.91%
		NGS	1886		
**crypt-based**	sulindac	Sanger	112	107	95.54%
		NGS	112		
	placebo	Sanger	120	115	95.83%
		NGS	120		

[#] Similarity: (Same pattern number/Total pattern number)×100%

Supplementary Table S5. Primers sequences of fusion PCR for NGS

Code	Sequence
	Forward Primers
**1F**	CCATCTCATCCCTGCGTGTCTCCGACTCAG**ACAGTATATA**CGATGTAAAACGACGGCCAG
**2F**	CCATCTCATCCCTGCGTGTCTCCGACTCAG**AGACTATACT**CGATGTAAAACGACGGCCAG
**3F**	CCATCTCATCCCTGCGTGTCTCCGACTCAG**AGTGCTACGA**CGATGTAAAACGACGGCCAG
**4F**	CCATCTCATCCCTGCGTGTCTCCGACTCAG**ACATACGCGT**CGATGTAAAACGACGGCCAG
**5F**	CCATCTCATCCCTGCGTGTCTCCGACTCAG**ACGACTACAG**CGATGTAAAACGACGGCCAG
**6F**	CCATCTCATCCCTGCGTGTCTCCGACTCAG**CTACGCTCTA**CGATGTAAAACGACGGCCAG
**7F**	CCATCTCATCCCTGCGTGTCTCCGACTCAG**TACACACACT**CGATGTAAAACGACGGCCAG
**8F**	CCATCTCATCCCTGCGTGTCTCCGACTCAG**TACGAGTATG**CGATGTAAAACGACGGCCAG
**9F**	CCATCTCATCCCTGCGTGTCTCCGACTCAG**TACTCTCGTG**CGATGTAAAACGACGGCCAG
**10F**	CCATCTCATCCCTGCGTGTCTCCGACTCAG**TACTGAGCTA**CGATGTAAAACGACGGCCAG
**11F**	CCATCTCATCCCTGCGTGTCTCCGACTCAG**TAGCATACTG**CGATGTAAAACGACGGCCAG
**12F**	CCATCTCATCCCTGCGTGTCTCCGACTCAG**CGACGTGACT**CGATGTAAAACGACGGCCAG
**13F**	CCATCTCATCCCTGCGTGTCTCCGACTCAG**CGCTCGAGTG**CGATGTAAAACGACGGCCAG
**14F**	CCATCTCATCCCTGCGTGTCTCCGACTCAG**ACGCGATCGA**CGATGTAAAACGACGGCCAG
**15F**	CCATCTCATCCCTGCGTGTCTCCGACTCAG**CACACGATAG**CGATGTAAAACGACGGCCAG
**16F**	CCATCTCATCCCTGCGTGTCTCCGACTCAG**CACTCGCACG**CGATGTAAAACGACGGCCAG
**17F**	CCATCTCATCCCTGCGTGTCTCCGACTCAG**AGTATACATA**CGATGTAAAACGACGGCCAG
**18F**	CCATCTCATCCCTGCGTGTCTCCGACTCAG**CAGTAGACGT**CGATGTAAAACGACGGCCAG
**19F**	CCATCTCATCCCTGCGTGTCTCCGACTCAG**TAGTCGCATA**CGATGTAAAACGACGGCCAG
**20F**	CCATCTCATCCCTGCGTGTCTCCGACTCAG**CTGCGTCACG**CGATGTAAAACGACGGCCAG
**21F**	CCATCTCATCCCTGCGTGTCTCCGACTCAG**TACTGAGCTA**CGATGTAAAACGACGGCCAG
**22F**	CCATCTCATCCCTGCGTGTCTCCGACTCAG**ATCACGTGCG**CGATGTAAAACGACGGCCAG
**23F**	CCATCTCATCCCTGCGTGTCTCCGACTCAG**CGTACTCAGA**CGATGTAAAACGACGGCCAG
**24F**	CCATCTCATCCCTGCGTGTCTCCGACTCAG**TCACGCGAGA**CGATGTAAAACGACGGCCAG
**25F**	CCATCTCATCCCTGCGTGTCTCCGACTCAG**TGATAGAGCG**CGATGTAAAACGACGGCCAG
**26F**	CCATCTCATCCCTGCGTGTCTCCGACTCAG**CGTGTCTCTA**CGATGTAAAACGACGGCCAG
	Reverse Primers
**1R**	GTCATAGCTGTTTCCTGATCG**AGTGTGTGT**ACTGAATCACCGTCTGCCCATAGAGAGG
**2R**	GTCATAGCTGTTTCCTGATCG**TAGTACGTG**ACTGAATCACCGTCTGCCCATAGAGAGG
**3R**	GTCATAGCTGTTTCCTGATCG**ACATACGTC**ACTGAATCACCGTCTGCCCATAGAGAGG
**4R**	GTCATAGCTGTTTCCTGATCG**TGTCGAGCGT**ACTGAATCACCGTCTGCCCATAGAGAGG
**5R**	GTCATAGCTGTTTCCTGATCG**TATACGATCG**ACTGAATCACCGTCTGCCCATAGAGAGG
**6R**	GTCATAGCTGTTTCCTGATCG**CGACAGATCG**ACTGAATCACCGTCTGCCCATAGAGAGG
**7R**	GTCATAGCTGTTTCCTGATCG**ACATAGTAGT**ACTGAATCACCGTCTGCCCATAGAGAGG
**8R**	GTCATAGCTGTTTCCTGATCG**CGCTGACGTG**ACTGAATCACCGTCTGCCCATAGAGAGG
**9R**	GTCATAGCTGTTTCCTGATCG**ACGCTATAG**ACTGAATCACCGTCTGCCCATAGAGAGG
**10R**	GTCATAGCTGTTTCCTGATCG**CTACAGTGCT**ACTGAATCACCGTCTGCCCATAGAGAGG

Bold letters in the middle show the different barcodes in each primer.

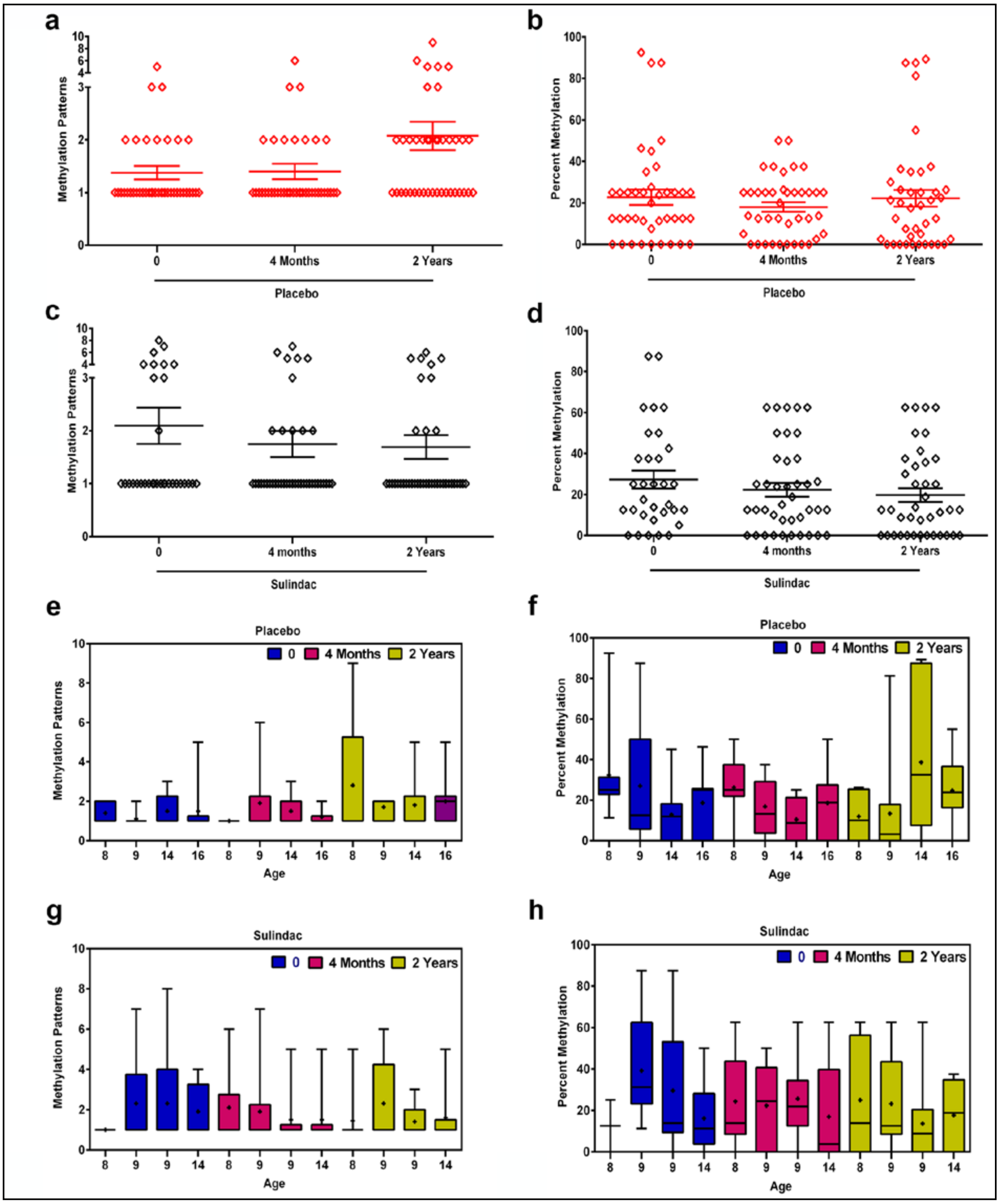

Supplementary Figure S1 Methylation patterns and percent methylation results of Sanger sequencing
